# Supplementary figures and images for: GWAS of QRS duration identifies new loci specific to Hispanic/Latino populations
Source: PLoS One. 2019 Jun 28;14(6):e0217796. doi: 10.1371/journal.pone.0217796 (PMC6599128; doi:10.1371/journal.pone.0217796)

**Supplementary Figure 1: QQ plot of Hispanic/Latino QRS duration GWAS meta-analysis**


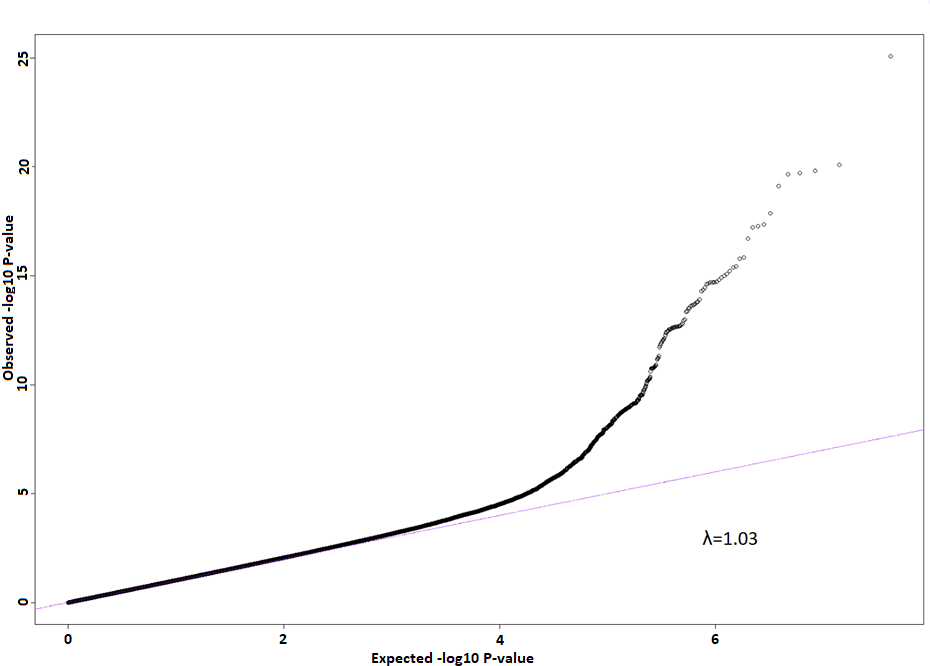

Supplement: S1 Fig — (DOCX) [file pone.0217796.s001.docx]

**Supplementary Figure 2: Individual Study QQ plots A) MESA B) HCHS/SOL C) WHI and D) Starr County**


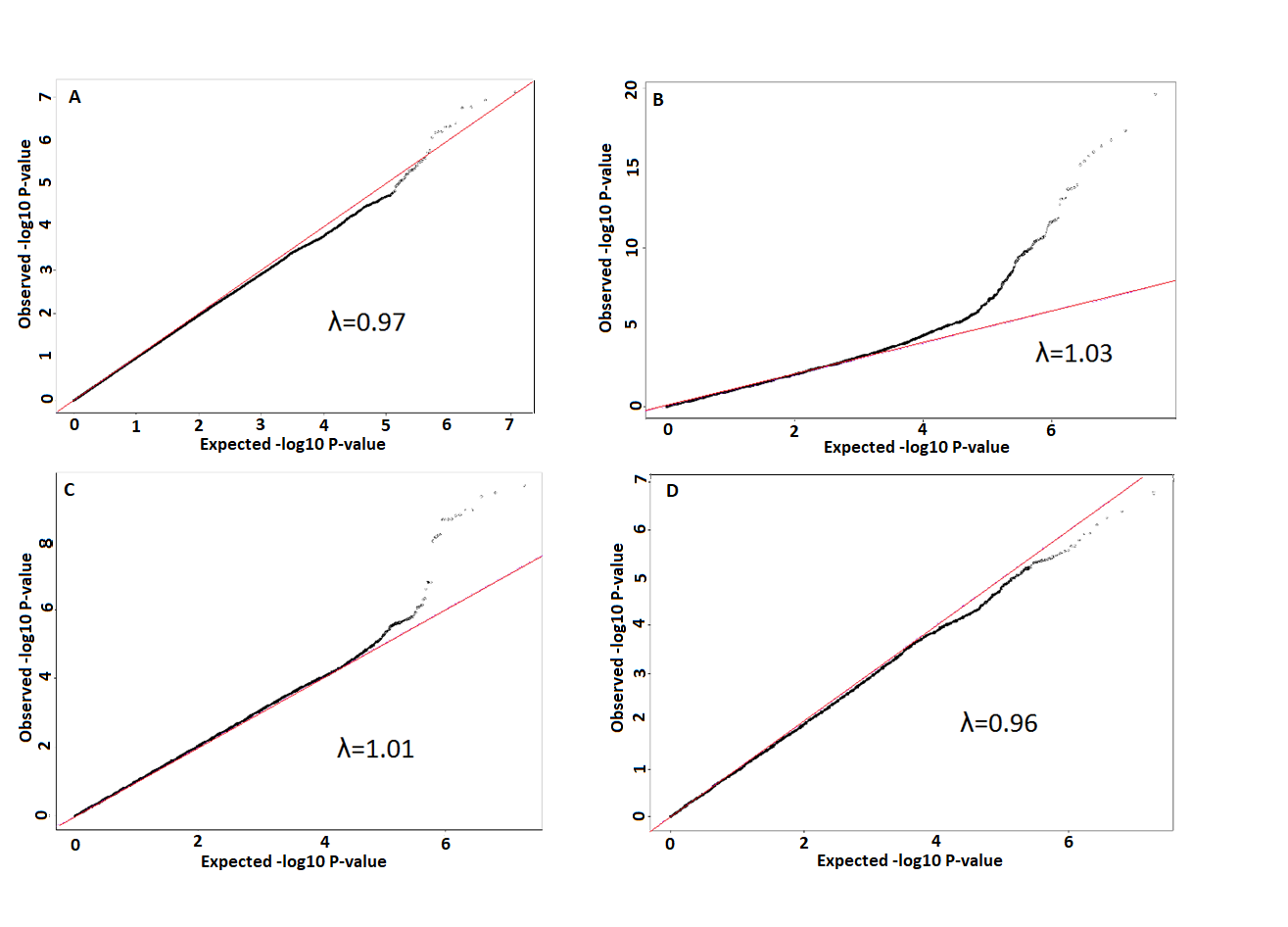

Supplement: S2 Fig — (DOCX) [file pone.0217796.s002.docx]
