# Supplementary material for: GWAS of QRS duration identifies new loci specific to Hispanic/Latino populations
Source: PLoS One. 2019 Jun 28;14(6):e0217796. doi: 10.1371/journal.pone.0217796 (PMC6599128; doi:10.1371/journal.pone.0217796)

**Supplementary Figure 3 - Forest plots showing 95% confidence intervals of the effect size of each Hispanic/Latino index SNP on QRS duration in milliseconds across each contributing study and the combined meta-analysis.**

**Supplementary Figure 3A**

**Index SNP: rs62241190**

**Effect Allele: G/A**


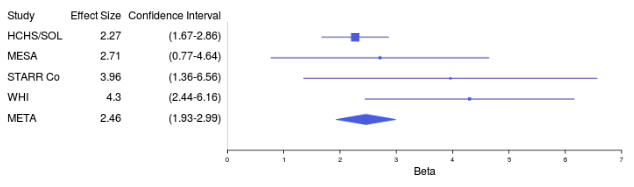


**Supplementary Figure 3B**

**Index SNP: rs3922844**

**Effect Allele: C/T**


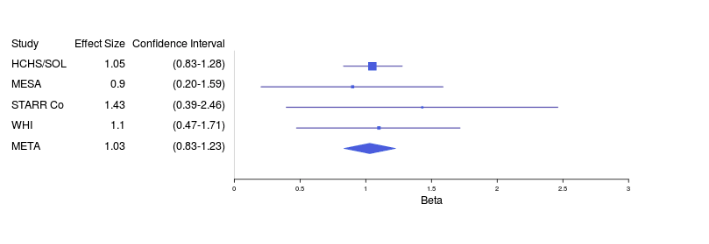


**Supplementary Figure 3C**

**Index SNP: rs9856387**

**Effect Allele: C/T**


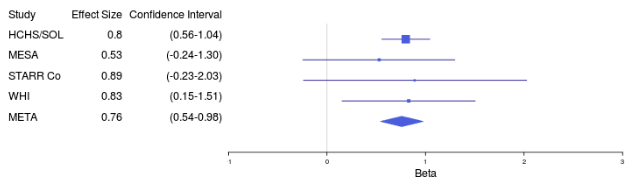


**Supplementary Figure 3D**

**Index SNP: rs10428132**

**Effect Allele: T/G**


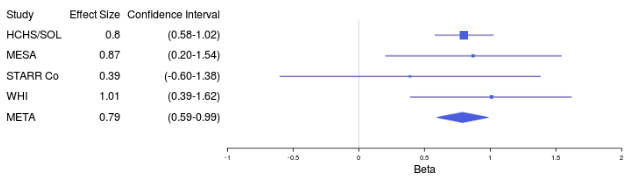


**Supplementary Figure 3E**

**Index SNP: rs13165478**

**Effect Allele: G/A**


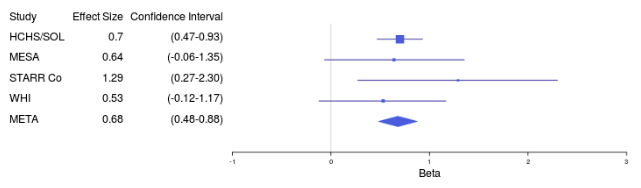


**Supplementary Figure 3F**

**Index SNP: rs3176326**

**Effect Allele: A/G**


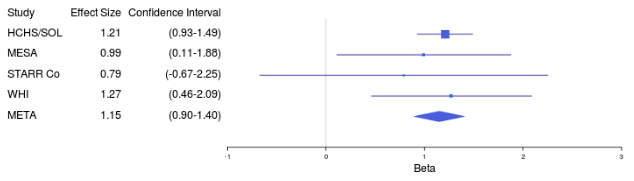


**Supplementary Figure 3G**

**Index SNP: rs2395642**

**Effect Allele: T/C**


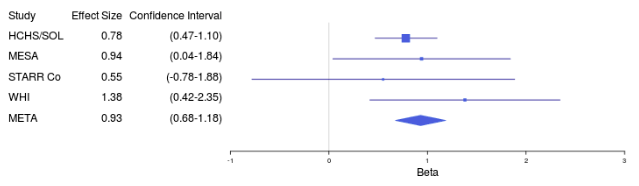


**Supplementary Figure 3H**

**Index SNP: rs7906312**

**Effect Allele: A/C**


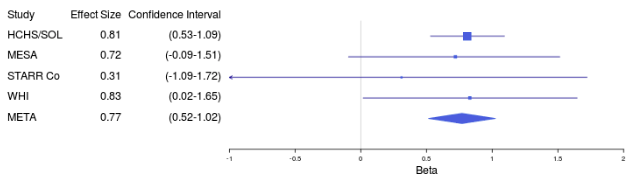


**Supplementary Figure 3I**

**Index SNP: rs4842438**

**Effect Allele: C/A**


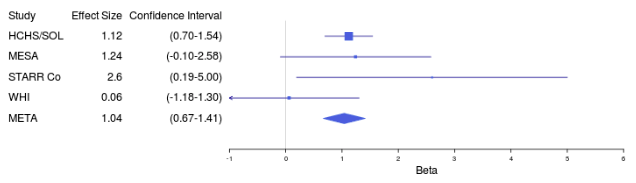


**Supplementary Figure 3J**

**Index SNP: rs16946539**

**Effect Allele: T/C**


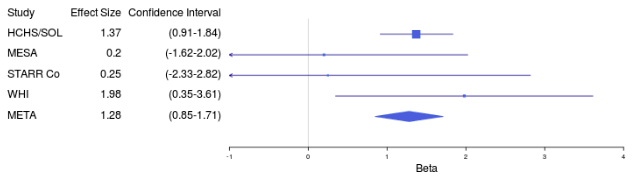

Supplement: S3 Fig — (DOCX) [file pone.0217796.s003.docx]
