# Supplementary material for: GWAS of QRS duration identifies new loci specific to Hispanic/Latino populations
Source: PLoS One. 2019 Jun 28;14(6):e0217796. doi: 10.1371/journal.pone.0217796 (PMC6599128; doi:10.1371/journal.pone.0217796)

**Supplementary Figure 4: Regional association plots showing all results in the European, Hispanic/Latino, and African American QRS duration GWAS surrounding each of the Hispanic/Latino significant loci.** Plots created with LocusZoom software.[21] The index SNP in each figure is labeled and colored purple. All other SNPs in the region are plotted at their significance levels. The color of each SNP corresponds to the linkage disequilibrium (r^2^) between the plotted SNP and the index SNP.

**Supplementary Figure 4A** – Regional association plots of *SCN5A-SCN10A*. **i.** (top-left) – Hispanic/Latino GWAS. **ii.** (top right) – European GWAS. **iii.** (bottom left) – African American GWAS


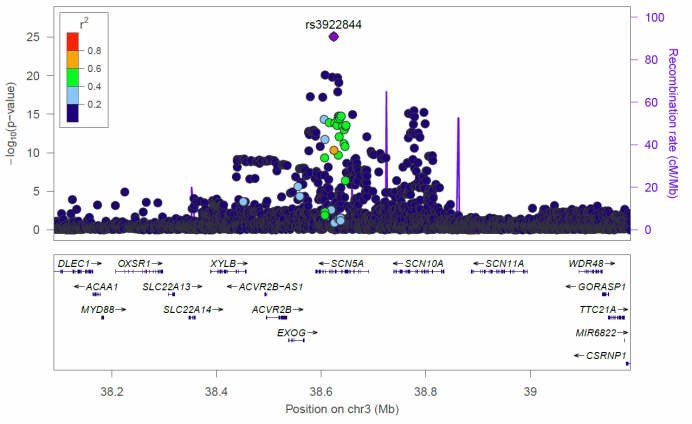

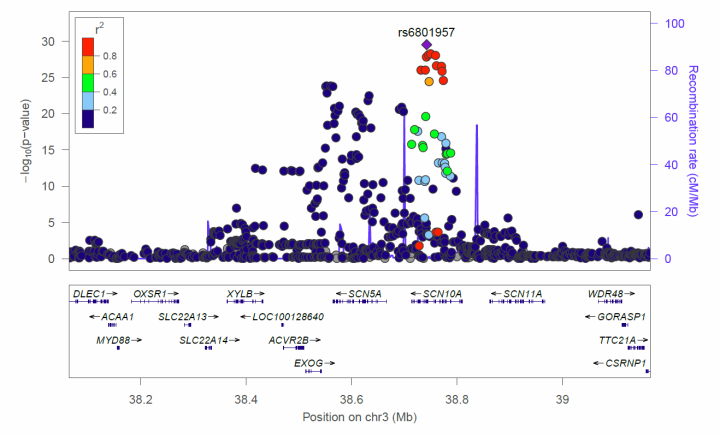

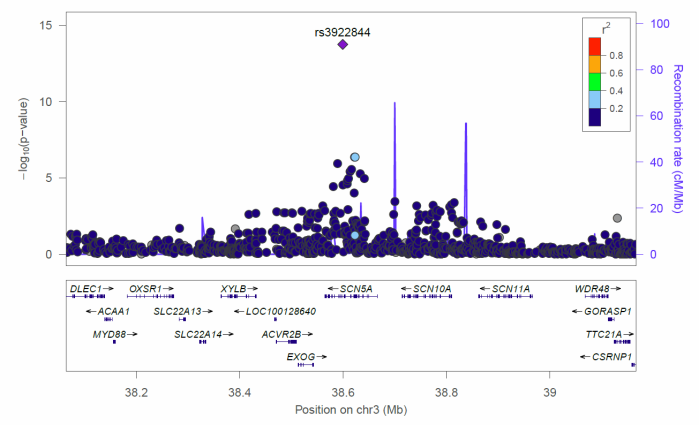


**Supplementary Figure 4B** – Regional association plots of *HAND1*. **i.** (top-left) – Hispanic/Latino GWAS. **ii.** (top right) – European GWAS. **iii.** (bottom left) – African American GWAS


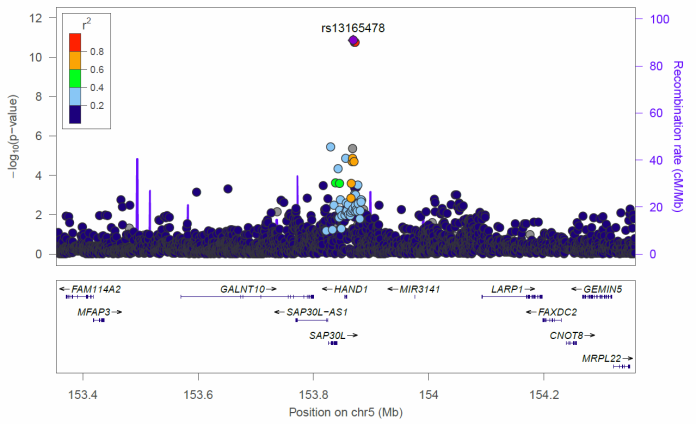

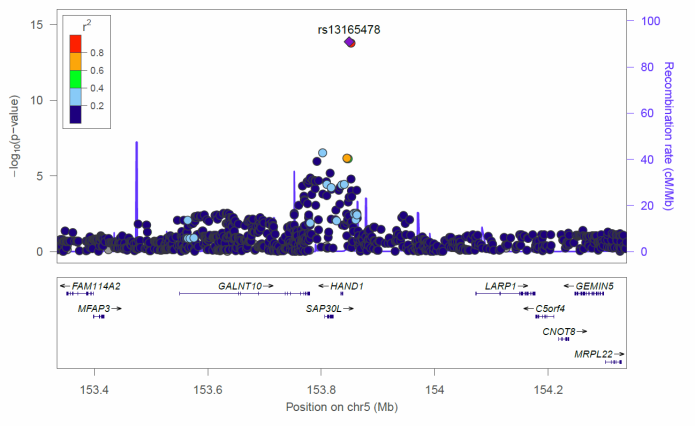


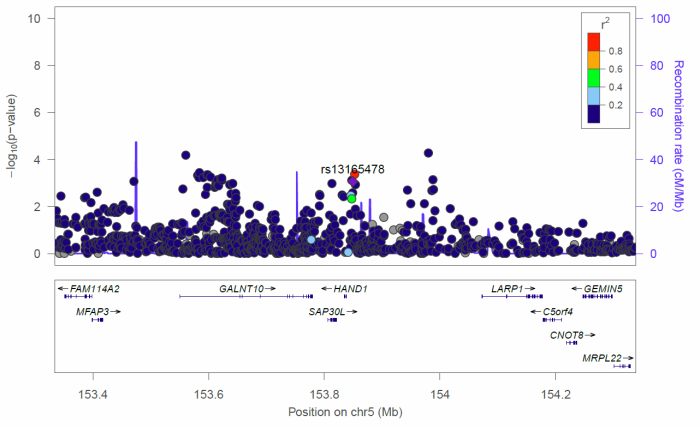


**Supplementary Figure 4C** – Regional association plots of *CDKN1A***. i.** (top-left) – Hispanic/Latino GWAS. **ii**. (top right) – European GWAS**. iii.** (bottom left) – African American GWAS**. iv.** (bottom right) – Hispanic/Latino GWAS showing an expanded view including SNPs near genome-wide significance.


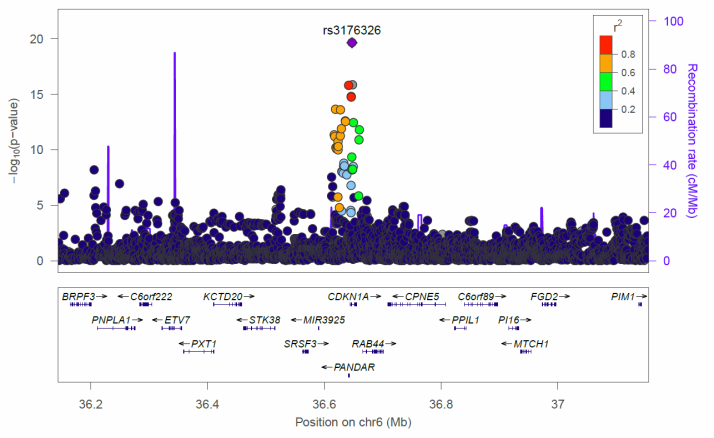

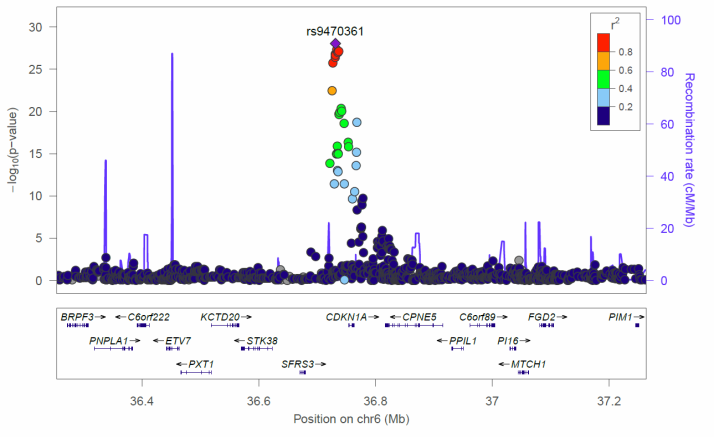


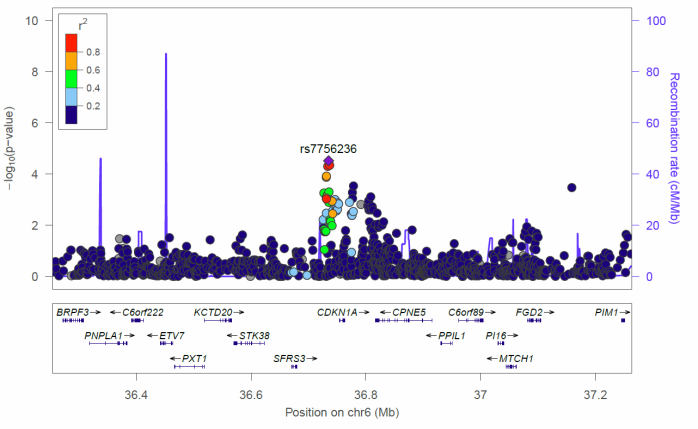

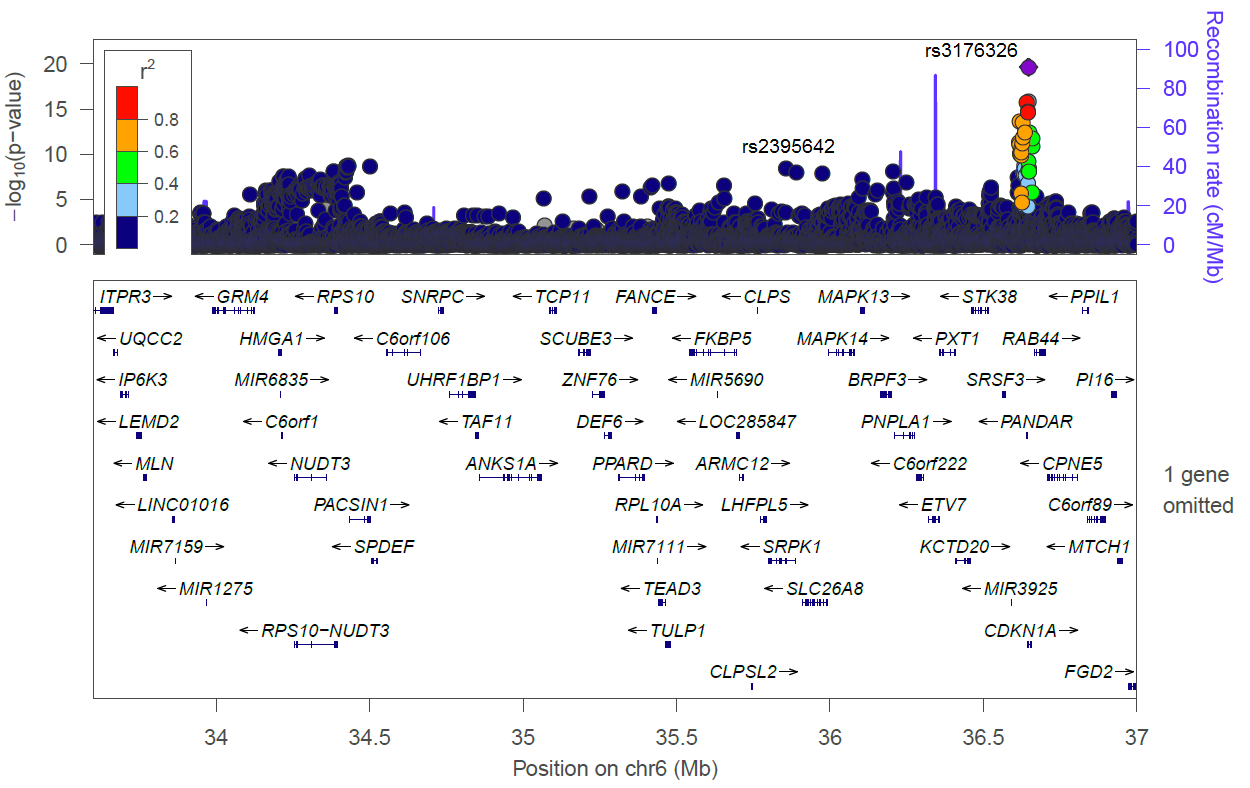


**Supplementary Figure 4D** – Regional association plots of *VTI1A*. **i.** (top-left) – Hispanic/Latino GWAS. **ii.** (top right) – European GWAS. **iii.** (bottom left) – African American GWAS


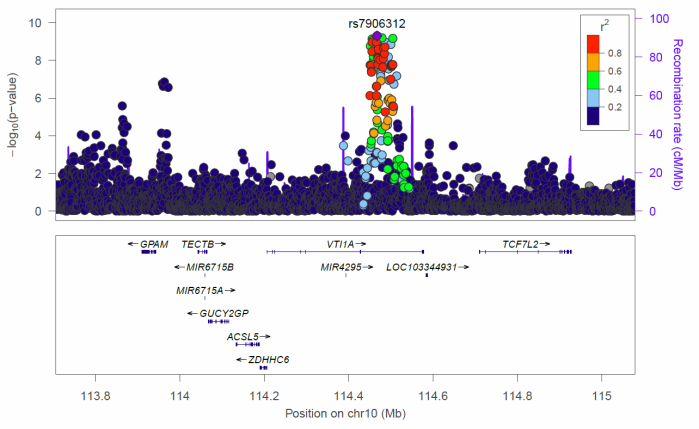

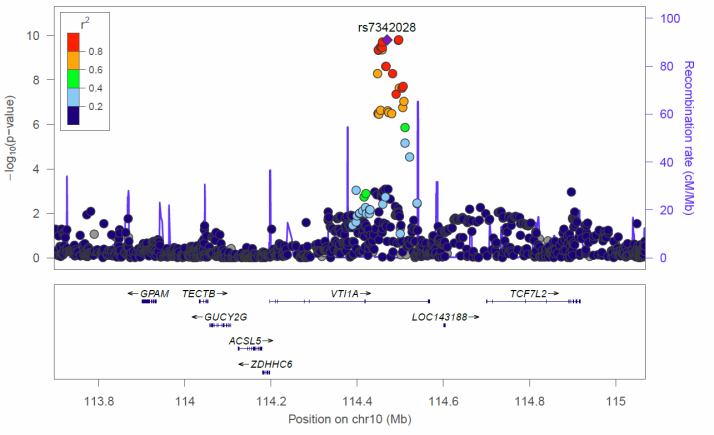


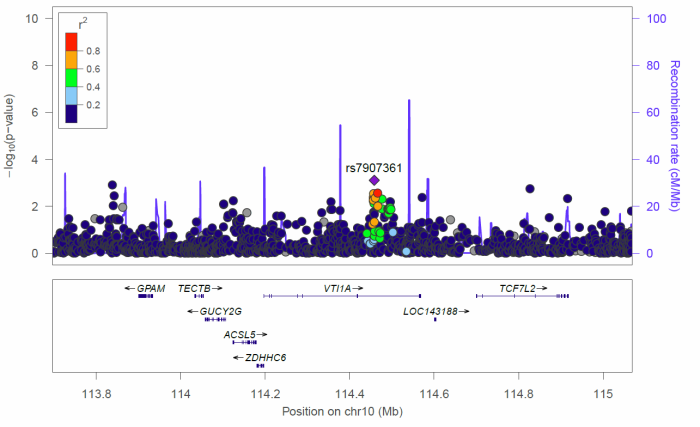


**Supplementary Figure 4E** – Regional association plots of *SYT1*. **i.** (top-left) – Hispanic/Latino GWAS. **ii.** (top right) – European GWAS. **iii.** (bottom left) – African American GWAS


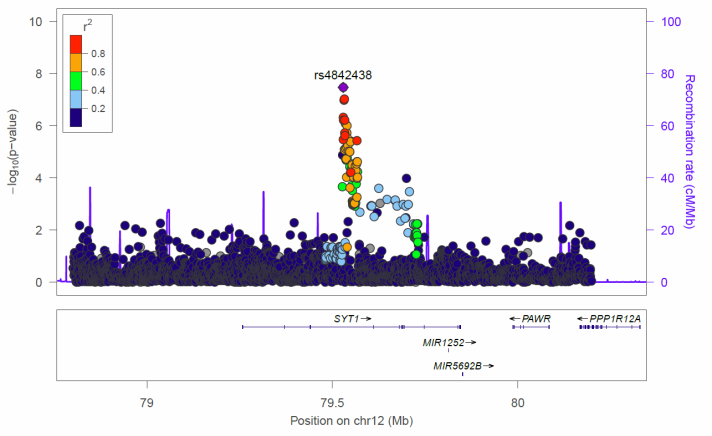

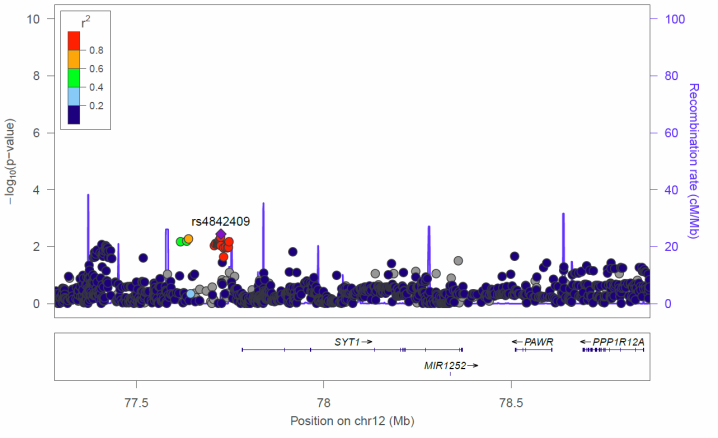


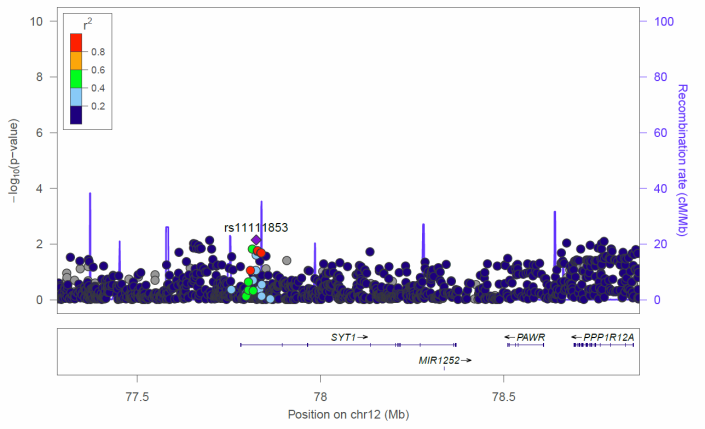


**Supplementary Figure 4F** – Regional association plots of *MYOCD*. **i.** (top-left) – Hispanic/Latino GWAS. **ii.** (top right) – European GWAS. **iii.** (bottom left) – African American GWAS


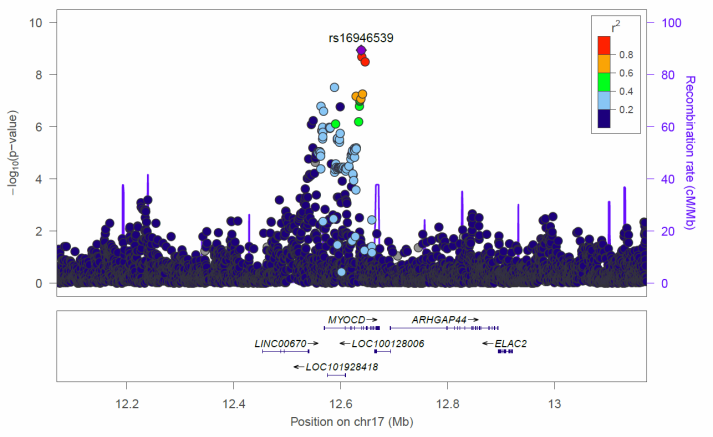

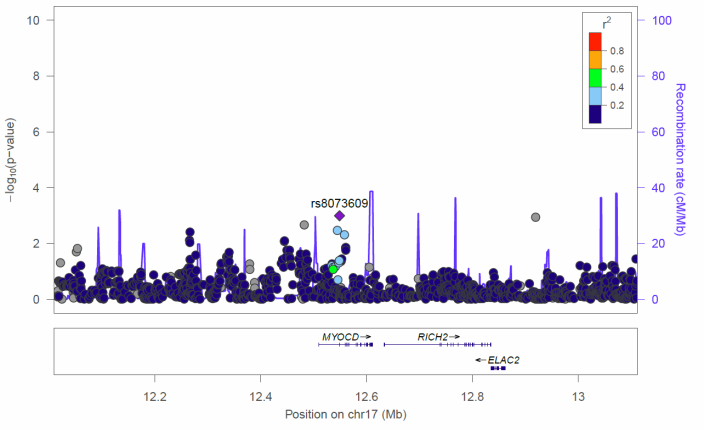


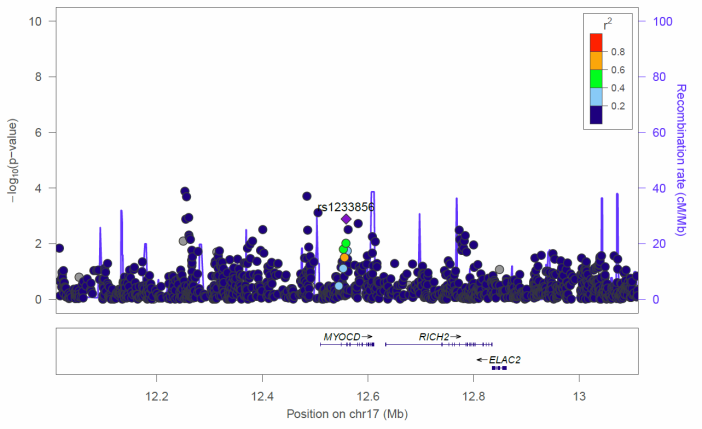

Supplement: S4 Fig — Plots created with LocusZoom software.[21] The index SNP in each figure is labeled and colored purple. All other SNPs in the region are plotted at their significance levels. The color of each SNP corresponds to the linkage disequilibrium (r2) between the plotted SNP and the index SNP. (DOCX) [file pone.0217796.s004.docx]
