# Supplementary material for: GWAS of QRS duration identifies new loci specific to Hispanic/Latino populations
Source: PLoS One. 2019 Jun 28;14(6):e0217796. doi: 10.1371/journal.pone.0217796 (PMC6599128; doi:10.1371/journal.pone.0217796)
Supplement: S5 Fig — Plots created with LocusZoom software.[21] Each plot shows all GWAS results surrounding the index SNP. The index SNP in each figure is labeled and colored purple. All other SNPs in the region are plotted at their significance levels. The color of each SNP corresponds to the linkage disequilibrium (r2) between the plotted SNP and the index SNP. SNPs plotted as circles were directly genotyped, and SNPs plotted as X’s were imputed. (DOCX) [file pone.0217796.s005.docx]

**Supplementary Figure 5: Regional association plots for the HCHS/SOL cohort for each of the index SNPs which was imputed rather than being directly genotyped in the cohort.** Plots created with LocusZoom software.[21] Each plot shows all GWAS results surrounding the index SNP. The index SNP in each figure is labeled and colored purple. All other SNPs in the region are plotted at their significance levels. The color of each SNP corresponds to the linkage disequilibrium (r^2^) between the plotted SNP and the index SNP. SNPs plotted as circles were directly genotyped, and SNPs plotted as X’s were imputed.

**Supplementary Figure 5A**

**Index SNP: rs2395642**

**Supplementary Figure 5B**

**Index SNP: rs3176326**

**Supplementary Figure 5C**

**Index SNP: rs4842438**

**Supplementary Figure 5D**

**Index SNP: rs16946539**

**Supplementary Figure 5E**

**Index SNP: rs62241190**
