# Supplementary material for: GWAS of QRS duration identifies new loci specific to Hispanic/Latino populations
Source: PLoS One. 2019 Jun 28;14(6):e0217796. doi: 10.1371/journal.pone.0217796 (PMC6599128; doi:10.1371/journal.pone.0217796)
Supplement: S1 Table — (DOCX) [file pone.0217796.s006.docx]

**Supplementary Table 1**. **Participant characteristics from the studies contributing to the meta-analysis.**

| **Characteristic** | **HCHS/SOL** | **MESA** | **Starr County** | **WHI** |
| --- | --- | --- | --- | --- |
| N | 11,566 | 1,431 | 582 | 1,545 |
| Sex, female % | 60.1 | 51.9 | 70.8 | 100.0 |
| Age, years mean (range) | 45 (18 – 76) | 61 (44 – 84) | 53 (32 – 87) | 60 (50 - 79) |
| QRS interval, ms, mean (SD) | 90.8 (9.5) | 91.1 (9.8) | 92.9 (15.3) | 85.5 (8.45) |
| BMI, kg/m^2^, mean | 29.68 | 29.51 | 32.19 | 29.34 |
| Hypertension, % | 25.66 | 41.93 | 42.47 | 29.88 |
| Diabetes mellitus, % | 18.25 | 17.8 | 45.6 | 7.5 |
| Heart rate, bpm, mean | 63 | 63 | 68 | 66 |
| Genomic control (λ_GC_) | 1.03 | 0.96 | 0.97 | 1.01 |
