# Supplementary material for: GWAS of QRS duration identifies new loci specific to Hispanic/Latino populations
Source: PLoS One. 2019 Jun 28;14(6):e0217796. doi: 10.1371/journal.pone.0217796 (PMC6599128; doi:10.1371/journal.pone.0217796)
Supplement: S2 Table — (DOCX) [file pone.0217796.s007.docx]

**Supplementary Table 2: Heterogeneity tests for index SNPs across the participating cohorts.**

| **Locus** | **Chr**^a^ | **Index SNP** | **Cochran’s Q Test** |
| --- | --- | --- | --- |
|  |  |  | ***P*** |
| *SCN5A* | 3 | rs62241190 | .144 |
| *SCN5A* | 3 | rs3922844 | .869 |
| *SCN5A* | 3 | rs9856387 | .919 |
| *SCN10A* | 3 | rs10428132 | .773 |
| *HAND1* | 5 | rs13165478 | .660 |
| *CDKN1A* | 6 | rs3176326 | .912 |
| *VTI1A* | 10 | rs7906312 | .917 |
| *SYT1* | 12 | rs4842438 | .241 |
| *MYOCD* | 17 | rs16946539 | .424 |

^a^Chr: Chromosome
