# Supplementary material for: GWAS of QRS duration identifies new loci specific to Hispanic/Latino populations
Source: PLoS One. 2019 Jun 28;14(6):e0217796. doi: 10.1371/journal.pone.0217796 (PMC6599128; doi:10.1371/journal.pone.0217796)
Supplement: S3 Table — (DOCX) [file pone.0217796.s008.docx]

**Supplementary Table 3: Summary of genetic imputation of index SNPs across the participating cohorts.**

| **Locus** | **Chr**^a^ | **Index SNP** | **HCHS/SOL** | **HCHS/SOL** | **MESA** | **MESA** | **Starr** | **Starr** | **WHI** | **WHI** |
| --- | --- | --- | --- | --- | --- | --- | --- | --- | --- | --- |
|  |  |  | **Imputation**^b^ | **Quality**^c^ | **Imputation**^b^ | **Quality**^c^ | **Imputation**^b^ | **Quality**^c^ | **Imputation**^b^ | **Quality**^c^ |
| *SCN5A* | 3 | rs62241190 | I | 0.951 | I | 0.772 | I | 0.856 | I | 0.992 |
| *SCN5A* | 3 | rs3922844 | G | 1 | I | 0.969 | I | 0.976 | I | 0.995 |
| *SCN5A* | 3 | rs9856387 | G | 1 | I | 0.919 | I | 0.943 | I | 0.984 |
| *SCN10A* | 3 | rs10428132 | G | 1 | I | 0.99264 | I | 0.996 | I | 0.997 |
| *HAND1* | 5 | rs13165478 | G | 1 | I | 0.954 | I | 0.989 | I | 0.995 |
| *CDKN1A* | 6 | rs3176326 | I | 0.983 | I | 0.886 | I | 0.913 | I | 0.989 |
| *VTI1A* | 10 | rs7906312 | G | 1 | I | 0.994 | I | 0.999 | I | 0.999 |
| *SYT1* | 12 | rs4842438 | I | 0.995 | I | 0.930 | I | 0.977 | I | 0.997 |
| *MYOCD* | 17 | rs16946539 | I | 0.999 | I | 0.775 | I | 0.791 | I | 0.987 |

^a^Chr: Chromosome

^b^Imputation: (G) Refers to a SNP which was directly typed on the assay. (I) Refers to a SNP which was imputed.

^c^Quality: Refers to the estimated probability that an average imputed genotype will match a genotype which was directly measured. This value was obtained via IMPUTE2 software[7] for HCHS/SOL, MESA, and Starr county cohorts, and via MaCH software[8,9] for WHI.
