# Supplementary material for: GWAS of QRS duration identifies new loci specific to Hispanic/Latino populations
Source: PLoS One. 2019 Jun 28;14(6):e0217796. doi: 10.1371/journal.pone.0217796 (PMC6599128; doi:10.1371/journal.pone.0217796)
Supplement: S4 Table — (DOCX) [file pone.0217796.s009.docx]

| **Supplementary Table 4. Coded allele frequencies for index SNPs significantly associated with QRS duration among participants of Hispanic/Latino ancestry (n=15,124).** | | | | | | | | |
| --- | --- | --- | --- | --- | --- | --- | --- | --- |
| **Locus** | **Index SNP** | **Chr**^a^ | **A1/A2**^b^ | **AFR**  **CAF**^c^ | **AMR**  **CAF**^d^ | **ASN**  **CAF**^e^ | **EUR**  **CAF**^f^ |  |
| *SCN5A* | rs62241190 | 3 | G/A | 0.00 | 0.03 | 0.00 | 0.02 |  |
| *SCN5A* | rs3922844 | 3 | C/T | 0.66 | 0.33 | 0.14 | 0.29 |  |
| *SCN5A* | rs9856387 | 3 | C/T | 0.73 | 0.69 | 0.77 | 0.67 |  |
| *SCN10A* | rs10428132 | 3 | T/G | 0.12 | 0.43 | 0.20 | 0.42 |  |
| *HAND1* | rs13165478 | 5 | G/A | 0.47 | 0.69 | 0.66 | 0.60 |  |
| *CDKN1A* | rs3176326 | 6 | A/G | 0.23 | 0.18 | 0.12 | 0.17 |  |
| *VTI1A* | rs7906312 | 10 | A/C | 0.61 | 0.12 | 0.38 | 0.03 |  |
| *SYT1* | rs4842438 | 12 | C/A | 0.80 | 0.93 | 0.94 | 0.94 |  |
| *MYOCD* | rs16946539 | 17 | T/C | 0.00 | 0.04 | 0.02 | 0.00 |  |
| ^a^Chr: Chromosome.  ^b^A1/A2: Coded/non-coded alleles.  ^c^AFR CAF: African 1000G Phase-1 super-population coding allele frequency obtained through HaploReg v4.[10]  ^d^AMR CAF: Ad Mixed American 1000G Phase-1 super-population coding allele frequency obtained through HaploReg v4.[10]  ^e^ASN CAF: East Asian 1000G Phase-1 super-population coding allele frequency obtained through HaploReg v4.[10]  ^f^EUR CAF: European 1000G Phase-1 super-population coding allele frequency obtained through HaploReg v4.[10] | | | | | | | | |
