# Supplementary material for: GWAS of QRS duration identifies new loci specific to Hispanic/Latino populations
Source: PLoS One. 2019 Jun 28;14(6):e0217796. doi: 10.1371/journal.pone.0217796 (PMC6599128; doi:10.1371/journal.pone.0217796)
Supplement: S6 Table — (DOCX) [file pone.0217796.s011.docx]

| **Supplementary Table 6: Index SNPs in Hispanic/Latino QRS duration GWAS (n=15,124) and corresponding SNPs in the African American QRS duration GWAS (n=13,301).[12]** | | | | | | | | | |
| --- | --- | --- | --- | --- | --- | --- | --- | --- | --- |
| **Hispanic/Latino Index SNP** | | | | **African American SNP in highest LD with Hispanic/Latino index SNP** | | | | | |
| **Locus** | **Gene** | **Index SNP** | **Chr**^a^ | **SNP** | **LD with Hispanic/Latino**  **Index SNP**^b^ | **A1/A2**^c^ | **African American CAF**^d^ | **African American β (ms)** | **African American *P*** |
| 1 | *SCN5A* | rs62241190 | 3 | rs2298422 | 0.20 | A/G | 0.01 | 0.47 | 1.53E-03 |
| 1 | *SCN5A* | rs3922844 | 3 | rs3922844 | 1.00 | C/T | 0.39 | 0.94 | 1.80E-14 |
| 1 | *SCN5A* | rs9856387 | 3 | rs6599234 | 0.15 | T/A | 0.70 | 0.35 | 3.32E-02 |
| 1 | *SCN10A* | rs10428132 | 3 | rs6599255 | 0.96 | A/C | 0.15 | 0.52 | 2.18E-03 |
| 2 | *HAND1* | rs13165478 | 5 | rs13165478 | 1.00 | G/A | 0.53 | 0.45 | 8.58E-04 |
| 3 | *CDKN1A* | rs3176326 | 6 | rs9462207 | 0.68 | C/T | 0.20 | 0.52 | 4.10E-04 |
| 4 | *VTI1A* | rs7906312 | 10 | rs7906312 | 1.00 | A/C | 0.52 | 0.32 | 7.58E-03 |
| 5 | *SYT1* | rs4842438 | 12 | rs4842438 | 1.00 | A/C | 0.19 | 0.00 | 9.76E-01 |
| 6 | *MYOCD* | rs16946539 | 17 | N/A (monomorphic) | - |  | - | - | - |

^a^Chr: Chromosome.

^b^LD: Linkage disequilibrium (r^2^).

^c^A1/A2: Coded/non-coded alleles.

^d^CAF: Coded allele frequency.
