# Supplementary material for: GWAS of QRS duration identifies new loci specific to Hispanic/Latino populations
Source: PLoS One. 2019 Jun 28;14(6):e0217796. doi: 10.1371/journal.pone.0217796 (PMC6599128; doi:10.1371/journal.pone.0217796)
Supplement: S7 Table — (DOCX) [file pone.0217796.s012.docx]

**Supplementary Table 7: Significant results for Welch’s t-tests of differences in effect sizes at Hispanic/Latino index SNPs across Hispanic/Latino, European, and African American GWAS results.**

| **SNP** | **Nearest**  **Gene** | **European vs Hispanic/Latino**  ***P***^a^ | **Hispanic/Latino vs African American *P***^a^ | **European vs African American**  ***P***^a^ |
| --- | --- | --- | --- | --- |
| rs17391905 | *C1orf185-RNF11-CDKN2C-FAF1* | **-** | **-** | 3.69E-04 |
| rs3922844 | *SCN5A* | 5.82E-05 | **-** | **-** |
| rs4687718 | *TKT-PRKCD- CACNA1D* | **-** | **-** | 3.97E-04 |
| rs4842438 | *SYT1* | 5.69E-05 | 8.65E-06 | **-** |

29 independent SNPs were examined for differences in effect sizes. With 3 separate tests per SNP (European vs Hispanic/Latino, Hispanic/Latino vs African American, and European vs African American) this resulted in 87 total tests (87=29x3). This yielded a Bonferroni corrected significance threshold set at 5.75E-04.

^a^*P* values are obtained through Welch’s t tests.
