# Supplementary material for: GWAS of QRS duration identifies new loci specific to Hispanic/Latino populations
Source: PLoS One. 2019 Jun 28;14(6):e0217796. doi: 10.1371/journal.pone.0217796 (PMC6599128; doi:10.1371/journal.pone.0217796)
Supplement: S8 Table — (DOCX) [file pone.0217796.s013.docx]

**Supplementary Table 8: Generalization of the associations in Hispanic/Latino QRS duration GWAS meta-analysis (n=15,124) for previously discovered loci from QRS duration GWAS among European (n=40,407),[11] African American, (n=13,301),[12] and East Asian (n=6805)[13] populations, and a European-African American meta-analysis (n=53,708).**

| **Locus** | **Chr**^a^ | **Index SNP** | **A1/A2**^b^ | **European** | **European** | **European** | **Hispanic/ Latino** | **Hispanic/**  **Latino** | **Hispanic/**  **Latino** | **Nearest** | **r-Value**^d^ |
| --- | --- | --- | --- | --- | --- | --- | --- | --- | --- | --- | --- |
|  |  |  |  | **CAF**^c^ | **GWAS β** | ***P*** | **CAF**^c^ | **GWAS β** | ***P*** | **Gene** |  |
| 1 | 1 | rs17391905 | T/G | 0.95 | 1.35 | 8.72E-09 | 0.98 | 1.08 | 1.33E-03 | *C1orf185-RNF11-CDKN2C-FAF1* | 4.56E-03 |
| 2 | 1 | rs9436640 | T/G | 0.54 | 0.59 | 4.57E-18 | 0.61 | 0.27 | 4.61E-03 | *NFIA* | 1.23E-02 |
| 3 | 1 | rs4074536 | T/C | 0.71 | 0.42 | 2.36E-08 | 0.64 | 0.36 | 2.39E-04 | *CASQ2* | 1.02E-03 |
| 4 | 2 | rs7562790 | G/T | 0.4 | 0.39 | 8.22E-09 | 0.45 | 0.29 | 1.85E-03 | *CRIM1* | 6.15E-03 |
| 5 | 2 | rs17020136 | C/T | 0.21 | 0.51 | 1.90E-09 | 0.26 | 0.26 | 1.01E-02 | *HEATR5B-STRN* | 2.34E-02 |
| 6 | 3 | rs2051211 | A/G | 0.74 | 0.44 | 1.57E-08 | 0.77 | 0.45 | 5.77E-05 | *EXOG* | 3.24E-04 |
|  | 3 | rs10865879 | C/A | 0.26 | 0.78 | 1.67E-24 | 0.20 | 0.87 | 5.24E-13 | *SCN5A/EXOG* | 1.34E-11 |
|  | 3 | rs11708996 | C/G | 0.16 | 0.79 | 1.26E-16 | 0.13 | 1.29 | 4.82E-19 | *SCN5A* | 1.18E-12 |
|  | 3 | rs11710077 | A/T | 0.79 | 0.84 | 5.72E-22 | 0.82 | 0.71 | 5.57E-09 | *SCN5A* | 7.30E-08 |
|  | 3 | rs9851724 | T/C | 0.67 | 0.66 | 1.91E-20 | 0.74 | 0.67 | 6.47E-10 | *SCN10A-SCN5A* | 9.53E-09 |
|  | 3 | rs6801957 | T/C | 0.41 | 0.77 | 1.10E-28 | 0.38 | 0.78 | 3.08E-15 | *SCN10A* | 2.40E-13 |
| 7 | 3 | rs4687718 | G/A | 0.86 | 0.63 | 6.25E-09 | 0.84 | 0.53 | 8.18E-05 | *TKT-PRKCD- CACNA1D* | 4.29E-04 |
| 8 | 3 | rs2242285 | A/G | 0.42 | 0.37 | 4.79E-08 | 0.34 | 0.27 | 4.30E-03 | *LRIG1-SLC25A26* | 1.17E-02 |
| 9 | 5 | rs13165478 | G/A | 0.64 | 0.55 | 7.36E-14 | 0.67 | 0.68 | 2.69E-11 | *HAND1-SAP30L* | 4.54E-10 |
| 10 | 6 | rs9470361 | A/G | 0.25 | 0.87 | 3.00E-27 | 0.22 | 0.76 | 3.46E-11 | *CDKN1A* | 5.34E-10 |
| 11 | 6 | rs11153730 | C/T | 0.49 | 0.59 | 1.26E-18 | 0.30 | 0.43 | 2.81E-05 | *C6orf204-SLC35F1-PLN-BRD7P3* | 1.72E-04 |
| 12 | 7 | rs1362212 | A/G | 0.18 | 0.69 | 1.12E-13 | 0.11 | 0.19 | 1.01E-01 | *TBX20* | 2.02E-01 |
| 13 | 7 | rs7784776 | G/A | 0.43 | 0.39 | 1.42E-08 | 0.48 | 0.21 | 1.64E-02 | *IGFBP3* | 3.61E-02 |
| 14 | 10 | rs1733724 | A/G | 0.25 | 0.49 | 1.26E-07 | 0.24 | 0.57 | 8.61E-07 | *DKK1* | 1.70E-04 |
| 15 | 10 | rs7342028 | T/G | 0.27 | 0.48 | 4.95E-10 | 0.32 | 0.42 | 4.09E-05 | *VTI1A* | 2.39E-04 |
| 16 | 12 | rs883079 | C/T | 0.29 | 0.49 | 1.33E-10 | 0.48 | 0.30 | 2.18E-03 | *TBX5* | 6.92E-03 |
| 17 | 12 | rs10850409 | G/A | 0.73 | 0.49 | 3.06E-10 | 0.61 | 0.25 | 6.38E-03 | *TBX3* | 1.54E-02 |
| 18 | 13 | rs1886512 | T/A | 0.63 | 0.40 | 4.31E-08 | 0.65 | 0.43 | 1.62E-05 | *KLF12* | 1.11E-04 |
| 19 | 14 | rs11848785 | A/G | 0.73 | 0.50 | 1.04E-10 | 0.83 | 0.31 | 7.99E-03 | *SIPA1L1* | 1.88E-02 |
| 20 | 17 | rs17608766 | C/T | 0.16 | 0.53 | 3.71E-07 | 0.07 | 0.64 | 4.33E-04 | *GOSR2* | 1.67E-03 |
| 21 | 17 | rs9912468 | G/C | 0.43 | 0.39 | 1.06E-08 | 0.42 | 0.42 | 8.28E-06 | *PRKCA* | 6.82E-05 |
| 22 | 18 | rs991014 | T/C | 0.42 | 0.42 | 6.20E-10 | 0.34 | 0.41 | 2.68E-05 | *SETBP1* | 1.70E-04 |
| **Locus** | **Chr**^a^ | **Index SNP** | **A1/A2** ^b^ | **African American** | **African American** | **African American** | **Hispanic/ Latino** | **Hispanic/**  **Latino** | **Hispanic/ Latino** | **Nearest** | **r-Value**^d^ |
|  |  |  |  | **CAF**^c^ | **GWAS β** | ***P*** | **CAF**^c^ | **GWAS β** | ***P*** | **Gene** |  |
| 6 | 3 | rs3922844 | C/T | 0.42 | 0.94 | 4.11E-14 | 0.63 | 1.03 | 1.19E-24 | *SCN5A* | 1.26E-22 |
| **Locus** | **Chr**^a^ | **Index SNP** | **A1/A2** ^b^ | **East Asian** | **East Asian** | **East Asian** | **Hispanic/ Latino** | **Hispanic/ Latino** | **Hispanic/ Latino** | **Nearest** | **r-Value**^d^ |
|  |  |  |  | **CAF**^c^ | **GWAS β** | ***P*** | **CAF**^c^ | **GWAS β** | ***P*** | **Gene** |  |
| 23 | 1 | rs2483280 | G/A | 0.72 | 0.68 | 1.51E-11 | 0.75 | 0.08 | 4.73E-01 | *PRDM16* | 6.76E-01 |
| 24 | 5 | rs335206 | C/T | 0.65 | 0.64 | 3.19E-10 | 0.35 | 0.35 | 2.48E-04 | *PRMD6* | 1.90E-03 |
| **Locus** | **Chr**^a^ | **Index SNP** | **A1/A2** ^b^ | **Meta** | **Meta** | **Meta** | **Hispanic/ Latino** | **Hispanic/ Latino** | **Hispanic/ Latino** | **Nearest** | **r-Value**^d^ |
|  |  |  |  | **CAF**^c^ | **GWAS β** | ***P*** | **CAF**^c^ | **GWAS β** | ***P*** | **Gene** |  |
| 25 | 1 | rs7547997 | A/G | 0.16 | 0.44 | 7.9E-09 | 0.19 | -0.07 | 5.32E-01 | *CD1E-OR10T2-SPTA1* | 7.95E-01 |
| 26 | 18 | rs1662342 | A/G | 0.14 | 0.47 | 4.9E-08 | 0.10 | 0.26 | 1.10E-01 | *MYL12A* | 2.26E-01 |

^a^Chr: Chromosome.

^b^A1/A2: Coded/non-coded alleles.

^c^CAF: Coded allele frequency

^d^r-Values are calculated via method described in *Sofer* et al.[14]
