# Supplementary material for: GWAS of QRS duration identifies new loci specific to Hispanic/Latino populations
Source: PLoS One. 2019 Jun 28;14(6):e0217796. doi: 10.1371/journal.pone.0217796 (PMC6599128; doi:10.1371/journal.pone.0217796)
Supplement: S9 Table — (DOCX) [file pone.0217796.s014.docx]

**Supplementary Table 9: Associations of QRS duration index SNPs with other ECG phenotypes (QT duration,[15] PR duration,[16] Heart Rate,[17] and Heart Rate Variability[17]) in the same Hispanic/Latino study population.**

| **Locus** | **Chr**^a^ | **Index SNP** | **A1/A2**^b^ | **QRS β (sd)**^c^ | **QRS *P*** | **QT β (sd)^c^** | **QT *P*** | **PR β (sd)**^c^ | **PR *P*** | **HR β (sd)**^d^ | **HR *P*** | **HRV β (sd)**^e^ | **HRV *P*** |
| --- | --- | --- | --- | --- | --- | --- | --- | --- | --- | --- | --- | --- | --- |
| *SCN5A* | 3 | rs62241190 | G/A | 2.46 (0.27) | 5.82E-20 | -3.58 (0.60) | 1.83E-09 | 4.72 (0.68) | 2.90E-12 | 0.06 (0.32) | 8.60E-01 | 0.02 (0.02) | 3.70E-01 |
| *SCN5A* | 3 | rs3922844 | C/T | 1.03 (0.10) | 1.19E-24 | -1.77 (0.22) | 9.52E-16 | 3.39 (0.25) | 3.57E-11 | -0.16 (0.12) | 1.82E-01 | 0.00 (0.01) | 8.26E-01 |
| *SCN5A* | 3 | rs9856387 | C/T | 0.76 (0.11) | 2.12E-12 | -1.42 (0.24) | 3.43E-09 | 1.88 (0.28) | 8.90E-12 | -0.18 (0.13) | 1.79E-01 | 0.00 (0.01) | 6.71E-01 |
| *SCN10A* | 3 | rs10428132 | T/G | 0.79 (0.10) | 1.43E-15 | -1.29 (0.22) | 3.04E-09 | 3.81 (0.25) | 4.89E-53 | -0.15 (0.12) | 2.04E-01 | -0.02 (0.01) | 8.10E-01 |
| *HAND1* | 5 | rs13165478 | G/A | 0.68 (0.10) | 2.69E-11 | 0.36 (0.23) | 1.10E-01 | -0.49 (0.26) | 6.05E-02 | 0.06 (0.12) | 6.44E-01 | 0.01 (0.01) | 2.79E-01 |
| *CDKN1A* | 6 | rs3176326 | A/G | 1.15 (0.13) | 1.54E-19 | -1.10 (0.28) | 8.59E-05 | 1.02 (0.32) | 1.44E-03 | -0.08 (0.15) | 5.97E-01 | 0.02 (0.01) | 8.19E-02 |
| *VTI1A* | 10 | rs7906312 | A/C | 0.77 (0.13) | 8.14E-10 | -0.09 (0.28) | 7.58E-01 | 0.22 (0.32) | 4.94E-01 | 0.01 (0.15) | 9.71E-01 | -0.02 (0.01) | 1.02E-01 |
| *SYT1* | 12 | rs4842438 | C/A | 1.04 (0.19) | 4.24E-08 | 0.51 (0.27) | 2.27E-01 | 0.27 (0.48) | 5.75E-01 | 0.14 (0.25) | 5.68E-01 | 0.00 (0.02) | 9.37E-01 |
| *MYOCD* | 17 | rs16946539 | T/C | 1.28 (0.22) | 1.74E-09 | 0.01 (0.48) | 9.81E-01 | -2.28 (0.53) | 1.82E-05 | 0.44 (0.26) | 9.00E-02 | -0.02 (0.02) | 3.57E-01 |

^a^Chr: Chromosome.

^b^A1/A2: Coded/non-coded alleles.

^c^β for QRS/QT/PR measured in milliseconds.

^d^β for heart rate (HR) measured in beats/minute

^e^β for heart rate variability (HRV) measured as the standard deviation of normal to normal R-R intervals, where R is the peak of the QRS complex (SDNN).
