# Supplementary material for: GWAS of QRS duration identifies new loci specific to Hispanic/Latino populations
Source: PLoS One. 2019 Jun 28;14(6):e0217796. doi: 10.1371/journal.pone.0217796 (PMC6599128; doi:10.1371/journal.pone.0217796)
Supplement: S10 Table — (DOCX) [file pone.0217796.s015.docx]

| **Supplementary Table 10**. **Summary of functional annotations in heart tissues for QRS duration significant loci in the HaploReg v4.1 database.[10]** | | | | |
| --- | --- | --- | --- | --- |
|  | | | | |
| **Locus** | **SNPs**^a^ | **Regulatory Motif^b^** | **Number of motifs** | **Tissues**^c^ |
| *SCN5A-SCN10A* | 3 | Enhancers | 6 | FH;RA;LV;RV |
| *CDKN1A* | 5 | Active TSS | 9 | FH;RA;LV;RV |
|  |  | Enhancers | 2 | FH |
|  |  | Flanking Active TSS | 1 | FH |
|  |  | Genic enhancers | 2 | RA |
| *MYOCD* | 3 | Enhancers | 8 | FH;RA;LV;RV |
| ^a^SNPs: number of SNPs in LD (r^2^ > 0.8) with any of the genome-wide associated SNPs including secondary signals in Table 1, with LD structure being determined by the AMR 1000 Genomes reference panel. Regulatory Motif: the chromatin state as defined by the 15-state model in ChromHMM algorithm.[18]  ^b^TSS: Transcription start site.  ^c^Tissues: FH, Fetal Heart; RA, Right Atrium; LV, Left Ventricle; RV Right Ventricle. | | | | |
