# Supplementary material for: GWAS of QRS duration identifies new loci specific to Hispanic/Latino populations
Source: PLoS One. 2019 Jun 28;14(6):e0217796. doi: 10.1371/journal.pone.0217796 (PMC6599128; doi:10.1371/journal.pone.0217796)
Supplement: S11 Table — (DOCX) [file pone.0217796.s016.docx]

**Supplementary Table 11. ECG and genotype measurement methods for the participating cohorts.**

| **Study** | **HCHS /SOL** | **MESA** | | **Starr County** | **WHI** |
| --- | --- | --- | --- | --- | --- |
| **ECG Measurements** | | | | | |
| ECG Machine | GE MAC 1200 | GE MAC 1200 |  | | Marquette MAC PC |
| ECG Measurement System | GE Marquette 12-SL | GE Marquette 12-SL software (2001 version) |  | | Marquette 12SL |
| **Genotype** | | | | | |
| Array | Illumina HumanOmni2.5-8v1-1 + custom content | Affymetrix Genome-Wide Human SNP Array 6.0 (Affymetrix, Santa Clara, CA) | Affymetrix 1751 (Affymetrix, Santa Clara, CA) | | Affymetrix Genome-Wide Human SNP Array 6.0 (Affymetrix, Santa Clara, CA) |
| Genotype calling software | GenomeStudio v2011.1 | Birdseed v1.33 | Birdseed and CRLMM concordant calls | | Birdseed |
| SNP call rate genotyping exclusion | <98% | <95% | <90% | | ≤95% |
| SNP MAF genotyping exclusion | NA | <1% | <1% | | <1% |
| SNP MAC genotyping exclusion | NA | NA | NA | | NA |
| *P* HWE genotyping exclusion | <1e-5 | <1e-6 | <1e-4 | | <1e-6 |
| MAF imputation exclusion | MAC >=2 in any of the 4 reference panels (AFR, AMR, ASN, EUR) | 0.01 | <0.05 | | NA |
| Imputation quality exclusion (IMPUTE2 INFO score or MaCH quality score) | NA | <0.4 | <0.8 | | NA |
| Imputation software | IMPUTE2 | IMPUTE2 | IMPUTE2 | | MaCH v1.0.16 |
| Build used for Imputation | 1000 Genomes Phase I release 3 (NCBI build 37 / hg19) | 1,000 Genomes Phase I integrated variant set (NCBI build 37 / hg19) | 1,000 Genomes Phase I integrated variant set (NCBI build 37 / hg19) | | 1,000 Genomes Phase I (NCBI Build 37/ hg19) |
| GWAS statistical analysis software | R/Bioconductor GENESIS package | SNPTEST2 | SNPTEST2 | | ProbAbel |
| Related Individuals? | Yes | No | Cleaned from analysis with PRIMUS[19,20] | | No |
| Familial Adjustment method | Kinship coefficients in Mixed model | NA | NA | | NA |
| # SNPs measured | 2,232,944 | 881666 | 603,042 | | 934,930 |
| # SNPSs imputed | 26x10^6^ | 39×10^6^ | 39×10^6^ | | 38×10^6^ |
| # SNPs passing QC | 17,322,742 | 8,637,954 | 5,997,534 | | 8,217,098 |
