# Supplementary material for: GWAS of QRS duration identifies new loci specific to Hispanic/Latino populations
Source: PLoS One. 2019 Jun 28;14(6):e0217796. doi: 10.1371/journal.pone.0217796 (PMC6599128; doi:10.1371/journal.pone.0217796)
Supplement: S1 Appendix — (DOCX) [file pone.0217796.s017.docx]

# **Description of Participating Studies**

*The Hispanic Community Health Study/Study of Latinos (HCHS/SOL)*

The HCHS/SOL is a multicenter, community-based cohort study of U.S. Hispanic/Latinos.[1,2] The goals of the study are to examine the prevalence of and risk factors for several disorders including heart, lung, blood, and kidney phenotypes. HCHS/SOL investigators sampled 16,415 males and females aged 18-74 years at baseline from four study communities: The Bronx, NY, Chicago, IL, Miami, FL, and San Diego, CA. HCHS/SOL recruitment centers were selected so that the study would include at least 2,000 participants in each of the following designations: Mexican, Puerto Rican, Dominican, Cuban, and Central and South American.

*Multi-Ethnic Study of Atherosclerosis (MESA)*

MESA was initiated in 2000 to investigate subclinical cardiovascular disease and the risk factors that predict progression to clinically overt cardiovascular disease.[3] The population-based cohort included 6,814 asymptomatic males and females aged 45–84 at study baseline from six field centers (Winston-Salem, NC; St. Paul, MN; Chicago, IL; Los Angeles, CA; New York, NY; Baltimore, MD). MESA investigators enrolled participants of Caucasian (38%), African American (28%), Hispanic (22%) and Chinese (12%) descent. The current study was restricted to Hispanic/Latino MESA participants who gave consent for DNA use. ECGs were measured at study baseline.

*Starr County*

These data were generated from examinations performed in follow-up from a systematic enumeration of 2507 households from 309 blocks selected randomly from the major population centers of Starr County, Texas. All subjects self-identified as Mexican American. Details of the participant examinations are previously described[4] as is generation, imputation, and quality control analyses of genotype data[5]. Echocardiography was among the measures taken; after sample exclusions 582 individuals remained in the analysis of QRS duration.

*Women’s Health Initiative Clinical Trial (WHI CT)*

The WHI comprises both randomized clinical trials (CT) and an observational study (OS). This study is limited to WHI CT participants, as ECGs were not collected for WHI OS participants.[6] The three WHI clinical trials were designed to allow randomized, controlled evaluation of 1) estrogen with or without progestin treatment, 2) calcium/vitamin D supplementation, and 3) dietary modification on the risk of breast and colorectal cancer, cardiovascular disease, and bone fractures. Between 1993 and 1998, the trials enrolled 68,132 postmenopausal women aged 50–79 years who were followed at 1 of 75 US examination sites (including satellites, remote sites, and their changes in location). Women were ineligible if they had medical conditions predictive of survival time less than 3 years, if they were known to have conditions inconsistent with study participation and adherence, or if they were active participants in another randomized, controlled trial. Those who remained eligible and interested were invited to follow-up examinations at 1, 3, 6, and 9 years. ECGs were measured at study baseline and analyses were restricted to African American and Hispanic/Latino WHI participants.

**Membership of GWAS Consortia used in Transethnic Analyses**

*CHARGE QRS GWAS Consortium* (European QRS GWAS)

Nona Sotoodehnia, Aaron Isaacs, Paul I. W. de Bakker, Marcus Dörr, Christopher Newton-Cheh, Ilja M. Nolte, Pim van der harst, Martina Müller, Mark Eijgelsheim, Alvaro Alonso, Andrew A. Hicks, Sandosh Padmanabhan, Caroline Hayward, Albert V. Smith, Ozren Polasek, Steven Giovannone, Jingyuan Fu, Jared W. Magnani, Kristin D. Marciante, Arne Pfeufer, Sina A. Gharib, Alexander Teumer, Man Li, Joshua C. Bis, Fernando Rivadeneira, Thor Aspelund, Anna Köttgen, Toby Johnson, Kenneth Rice, Mark P. S. Sie, Ying A. Wang, Norman Klopp, Christian Fuchsberger, Sarah H. Wild, Irene M. Leach, Karol Estrada, Uwe Völker, Alan F. Wright, Folkert W. Asselbergs, Jiaxiang Qu, Aravinda Chakravarti, Moritz F. Sinner, Jan A. Kors, Astrid Petersmann, Tamara B. Harris, Elsayed Z. Soliman, Patricia B. Munroe, Bruce M. Psaty, Ben A. Oostra, L. Adrienne Cupples, Siegfried Perz, Rudolf A. de Boer, André G. Uitterlinder, Henry Völzke, Timothy D. Spector, Fang-Yu Liu, Eric Boerwinkle, Anna F. Dominiczak, Jerome I. Rotter, Gé van Herpen, Daniel Levy, H-Erich Wichmann, Wiek H. van Gilst, Jacqueline C. M. Witteman, Heyo K. Kroemer, W. H. Linda Kao, Susan R. Heckbert, Thomas Meitinger, Albert Hofman, Harry Campbell, Aaron R. Folsom, Dirk J. van Veldhuisen, Christine Schwienbacher, Christopher J. O’Donnell, Claudia B. Volpato, Mark J. Caulfield, John M. Connell, Lenore Launer, Xiaowen Lu, Lude Franke, Rudolf S. N. Fehrmann, Gerard te Meerman, Harry J. M. Groen, Rinse K. Weersma, Leonard H. van den Berg, Cisca Wiljmenga, Roel A. Ophoff, Gerjan Navis, Igor Rudan, Harold Sneider, James F. Wilson, Peter P. Pramstaller, David S. Siscovick, Thomas J. Wang, Vilmundur Gudnason, Cornelia M. van Duijn, Stephan B. Felix, Glenn I Fishman, Yalda Jamshidi, Bruno H. Ch Stricker, Nilesh J. Samani, Stefan Kääb, and Dan E. Arking.

*CARe-COGENT African-American QRS Consortium*

Daniel S. Evans, Christy L. Avery, Mike A. Nalls, Guo Li, John Barnard, Erin N. Smith, Toshiko Tanaka, Anne M. Butler, Sarah G. Buxbaum, Alvaro Alonso, Dan E. Arking, Gerald S. Berenson, Joshua C. Bis, Steven Buyske, Cara L. Carty, Wei Chen, Mina K. Chung, Steven R. Cummings, Rajat Deo, Charles B. Eaton, Ervin R. Fox, Susan R. Heckbert, Gerardo Heiss, Lucia A. Hindorff, Wen-Chi Hsueh, Aaron Isaacs, Yalda Jamshidi, Kathleen F. Kerr, Felix Liu, Yongmei Liu, Kurt K. Lohman, Jared W. Magnani, Joseph F. Maher, Reena Mehra, Yan A. Meng, Solomon K. Musani, Christopher Newton-Cheh, Kari E. North, Bruce M. Psaty, Susan Redline, Jerome I. Rotter, Renate B. Schnabel, Nicholas J. Schork, Ralph V. Shohet, Andrew B. Singleton, Jonathan D. Smith, Elsayed Z. Soliman, Sathanur R. Srinivasan, Herman A. Taylor, Jr., David R. Van Wagoner, James G. Wilson, Taylor Young, Zhu-Ming Zhang, Alan B. Zonderman, Michele K. Evans, Luigi Ferrucci, Sarah S. Murray, Gregory J. Tranah, Eric A. Whitsel, Alex P. Reiner, Nona Sotoodehnia.

**References**

1. LaVange LM, Kalsbeek WD, Sorlie PD, Avilés-Santa LM, Kaplan RC, Barnhart J, et al. Sample design and cohort selection in the Hispanic Community Health Study/Study of Latinos. Ann Epidemiol. 2010;20(8):642-9.

2. Sorlie PD, Avilés-Santa LM, Wassertheil-Smoller S, Kaplan RC, Daviglus ML, Giachello AL, et al. Design and implementation of the Hispanic community health study/study of Latinos. Ann Epidemiol. 2010;20(8):629-41.

3. Bild DE, Bluemke DA, Burke GL, Detrano R, Roux AVD, Folsom AR, et al. Multi-ethnic study of atherosclerosis: objectives and design. Am J Epidemiol. 2002;156(9):871-81.

4. Hanis CL, Redline S, Cade BE, Bell GI, Cox NJ, Below JE, et al. Beyond type 2 diabetes, obesity and hypertension: an axis including sleep apnea, left ventricular hypertrophy, endothelial dysfunction, and aortic stiffness among Mexican Americans in Starr County, Texas. Cardiovasc Diabetol 2016;15(1):1.

5. Below JE, Parra EJ, Gamazon ER, Torres J, Krithika S, Candille S, et al. Meta-analysis of lipid-traits in Hispanics identifies novel loci, population-specific effects, and tissue-specific enrichment of eQTLs. Sci Rep. 2016;6:19429.

6. Anderson G, Cummings S, Freedman L, Furberg C, Henderson M, Johnson S, et al. Design of the Women's Health Initiative clinical trial and observational study. Control Clin Trials. 1998;19(1):61-109.

7. Howie BN, Donnelly P, Marchini J. A flexible and accurate genotype imputation method for the next generation of genome-wide association studies. PLoS Genet. 2009;5(6):e1000529.

8. Li Y, Willer C, Sanna S, Abecasis G. Genotype imputation. Annu Rev Genomics Hum Genet. 2009;10:387-406.

9. Li Y, Willer CJ, Ding J, Scheet P, Abecasis GR. MaCH: using sequence and genotype data to estimate haplotypes and unobserved genotypes. Genetic Epidemiol. 2010;34(8):816-34.

10. Ward LD, Kellis M. HaploReg v4: systematic mining of putative causal variants, cell types, regulators and target genes for human complex traits and disease. Nucleic Acids Res. 2016;44(D1):D877-D81.

11. Sotoodehnia N, Isaacs A, de Bakker PI, Dörr M, Newton-Cheh C, Nolte IM, et al. Common variants in 22 loci are associated with QRS duration and cardiac ventricular conduction. Nat Genet. 2010;42(12):1068-76.

12. Evans DS, Avery CL, Nalls MA, Li G, Barnard J, Smith EN, et al. Fine-mapping, novel loci identification, and SNP association transferability in a genome-wide association study of QRS duration in African Americans. Hum Mol Genet. 2016:;ddw284.

13. Hong K-W, Lim JE, Kim JW, Tabara Y, Ueshima H, Miki T, et al. Identification of three novel genetic variations associated with electrocardiographic traits (QRS duration and PR interval) in East Asians. Hum Mol Genet. 2014;23(24):6659-67.

14. Sofer T, Heller R, Bogomolov M, Avery CL, Graff M, E. NK, et al. A powerful statistical framework for generalization testing in GWAS, with application to the HCHS/SOL. Genet Epidemiol. 2017;41(3):251-8.

15. Méndez-Giráldez R, Gogarten SM, Below JE, Yao J, Seyerle AA, Highland HM, et al. GWAS of the electrocardiographic QT interval in Hispanics/Latinos generalizes previously identified loci and identifies population-specific signals. Sci Rep. 2017;7(1):17075.

16. Seyerle AA, Lin HJ, Gogarten SM, Stilp A, Giráldez RM, Soliman E, et al. Genome-wide association study of PR interval in Hispanics/Latinos identifies novel locus at ID2. Heart. 2018;104(11):904-11.

17. Kerr KF, Avery CL, Lin HJ, Raffield LM, Zhang QS, Browning BL, et al. Genome-wide association study of heart rate and its variability in Hispanic/Latino cohorts. Heart Rhythm. 2017;14(11):1675-84.

18. Ernst J, Kellis M. ChromHMM: automating chromatin-state discovery and characterization. Nat Methods. 2012;9(3):215.

19. Staples J, Nickerson DA, Below JE. Utilizing graph theory to select the largest set of unrelated individuals for genetic analysis. Genet Epidemiol. 2013;37(2):136-41.

20. Staples J, Qiao D, Cho MH, Silverman EK, Nickerson DA, Below JE, et al. PRIMUS: rapid reconstruction of pedigrees from genome-wide estimates of identity by descent. Am J Hum Genet. 2014;95(5):553-64.

21. Pruim RJ, Welch RP, Sanna S, Teslovich TM, Chines PS, Gliedt TP, et al. LocusZoom: regional visualization of genome-wide association scan results. Bioinformatics. 2010;26(18):2336-7.
